# Ultrasonographic findings in long COVID: A cross-sectional study of 312 patients

**DOI:** 10.3389/fmed.2022.1051389

**Published:** 2023-01-09

**Authors:** Marta Imamura, Sabrina Saemy Tome Uchyiama, Gabriella Souza Naves, Cláudia Andréia Rabay Pimentel Abicalaf, Aline Rossetti Mirisola, Artur César Aquino dos Santos, Linamara Rizzo Battistella, HCFMUSP COVID-19 Study Group

**Affiliations:** ^1^Departamento de Medicina Legal, Bioética, Medicina do Trabalho e Medicina Física e Reabilitação, Faculdade de Medicina da Universidade de São Paulo, São Paulo, Brazil; ^2^Instituto de Medicina Física e Reabilitação, Hospital das Clinicas da Faculdade de Medicina da Universidade de São Paulo, São Paulo, Brazil; ^3^Hospital das Clínicas da Faculdade de Medicina da Universidade de São Paulo, São Paulo, Brazil

**Keywords:** long covid, diagnostic imaging, ultrasonography, muscle thickness, echo-intensity

## Abstract

**Background:**

Fatigue and muscle weakness are common complaints in COVID-19 survivors. However, little is still known about the skeletal muscle qualitative and quantitative characteristics after hospitalization due to moderate and severe COVID-19.

**Objectives:**

To assess *rectus femoris* and *vastus intermedius* muscle thickness (MT) and *rectus femoris* echo intensity (EI) and to establish its association with demographic, clinical, functional, and inflammatory parameters in long COVID patients after hospital discharge.

**Methods:**

Cross-sectional study with 312 COVID-19 patients (53.53% male; age: 54.59 ± 13.50 years), with a laboratory-confirmed diagnosis of COVID-19. Patients were assessed 3–11 months after hospital discharge. We evaluated MT of the right *rectus femoris* and v*astus intermedius* and EI of the right *rectus femoris* using a portable ultrasound system, 6–13 MHz, broadband linear transducer. We corrected EI using the subcutaneous fat thickness. Ultrasonographic parameters were tested in association with demographic (sex and age); functional (Handgrip strength measurement, Timed Up and Go, 1 min Sit-to-Stand test, EuroQoL-5 Dimensions-5 Levels, World Health Organization Disability Assessment Schedule (WHODAS 2.0), Post-COVID-19 Functional Status, Functional Assessment of Chronic Illness Therapy—Fatigue (FACIT), Medical Research Council (MRC) sum score, Borg Dyspnea Scale, MRC Dyspnea score, Visual Analogue Scale (VAS), Epworth Sleepiness Scale, Insomnia Severity Index, Functional Independence Measurement (FIM), and Functional Oral Intake Scale); clinical (length of hospital stay, intubation, and presence of comorbidities such as systemic hypertension, diabetes, obesity, chronic obstructive pulmonary disease, asthma), and inflammatory data assessed by the C-reactive protein and D-dimer serum concentrations.

**Results:**

*Rectus femoris* MT was associated with age, handgrip strength, Epworth Sleepiness Scale, and subcutaneous fat thickness (r^2^ = 27.51%; *p* < 0.0001). *Vastus intermedius* MT was associated with age, pain intensity, handgrip strength, Epworth Sleepiness scale, FIM, and time since hospital discharge (r^2^ = 21.12%; *p* < 0.0001). *Rectus femoris* EI was significantly associated with the male sex, TUG, Epworth Sleepiness Scale, and C-Reactive Protein levels (r^2^ = 44.39%; *p* < 0.0001). Mean MT of *rectus femoris* and *vastus intermedius* are significantly different (*p* < 0.001).

**Conclusion:**

After hospital discharge, long COVID patients present qualitative and quantitative skeletal muscle characteristics associated with a combination of demographic, clinical, and functional parameters.

## 1. Introduction

The World Health Organization defines long COVID as a condition that occurs in individuals with a history of probable or confirmed SARS-CoV-2 infection, usually 3 months from the onset of COVID-19 with symptoms that last for at least 2 months, that cannot be explained by an alternative diagnosis (1). Among the several complaints described in long COVID patients, increased fatigue and muscle weakness are prevalent symptoms at different follow-up periods after hospital discharge (2–5). Muscle atrophy, diminished muscle tension, infiltration of fat and/or fibrosis within the skeletal muscle fibers, and changes in the neuromuscular activation may be involved in the multifactorial and complex reasons for muscle weakness in long COVID patients (6). Both qualitative and quantitative changes in the skeletal muscle morphology may occur at different levels and in different long COVID patients.

Several potential factors can modify muscle morphology in patients with long COVID (6). SARS-Cov-2 can invade the skeletal muscle cells by binding to its angiotensin-converting enzyme 2 (ACE2) receptors causing muscle weakness and atrophy (6, 7). Increased circulating levels of pro-inflammatory cytokines may also influence muscle loss in long COVID patients (8) by negatively impacting muscle protein metabolism. Other possible mechanisms of muscle loss include decreased muscle protein synthesis and increased muscle protein breakdown (8). Sarcopenic obesity due to adipose tissue accumulation is another component to be better assessed in COVID-19 survivors (8). Physical inactivity and increased sedentary lifestyles exacerbated by quarantine and social reclusion measures during the first 2 years of the pandemic and poor nutritional status after hospital discharge may have negatively affected skeletal muscle morphology in long COVID patients (6). Previous pre-existing sarcopenia may be another cause of muscle weakness in aged long COVID patients (6). Intensive care unit (ICU)-acquired muscle weakness may sustain physical disability in the long term (6). Fat, fibrosis infiltration, and a selective loss of myosin may change muscle quality and contribute to muscle weakness (6). In summary, systemic inflammation (8), viral infiltration (6, 7), muscle disuse (6), hypoxemia, malnutrition, and the adverse effects of medications are factors contributing to muscle weakness and exercise intolerance in patients with COVID-19.

Despite available evidence of fatigue and muscle weakness as primary symptoms in long COVID patients, to the best of our knowledge, there is still no described assessment of their muscle architecture and quality. B mode Ultrasound (US) evaluation already depicted progressive *rectus femoris* and *vastus intermedius* muscle wasting in severely affected COVID-19 patients during the first 7 to 10 days of intensive care unit stay (9, 10). However, it is still unknown whether these findings are also present in long COVID patients discharged from the hospital to treat the acute infection.

Musculoskeletal US is a non-invasive, fast, valid, practical, and safe imaging technique that measures muscle thickness (MT) and echo-intensity (EI) in the daily routine of an ICU (11, 12) and other health facilities (13). This technique is reliable (ICC ≥ 0.902, SEMs ≤ 5.01% (14, 15) and consistent regardless of different assessors (16–18). Musculoskeletal US measurements seem to be as accurate as those of resonance imaging (19, 20), and Dual-energy X-ray absorptiometry (DXA) (21), the gold standard for assessing muscle mass (18, 21). Simultaneous reports of quantitative (MT) and qualitative (EI) allow a more comprehensive assessment of muscle characteristics using a no radiation and affordable tool to assess muscle morphology.

Therefore, given the lack of comprehension regarding muscle morphology of individuals who endure post-acute SARS-CoV-2 sequelae, we assessed US to quantify MT and EI in hospitalized long COVID patients after hospital discharge. We have also established the association of *rectus femoris* and *vastus intermedius* MT and *rectus femoris* EI with demographic, clinical, and functional outcomes of COVID-19 survivors. Our objective was to assess *rectus femoris* and *vastus intermedius* MT and *rectus femoris*, EI and establish associations with demographic, clinical, and functional outcomes of hospitalized COVID-19 survivors.

We hypothesize that long COVID patients hospitalized for acute infection management may still present the skeletal muscle morphological characteristics demonstrated during hospitalization in the intensive care unit (9, 10).

## 2. Materials and methods

### 2.1. Study population

We conducted cross-sectional analyses on 312 patients, 18 years or older, who were hospitalized at HCFMUSP (*Hospital das Clínicas da Faculdade de Medicina da Universidade de São Paulo*) for more than 24 h between March and August 2020, with a diagnosis of COVID-19 confirmed by either Polymerase Chain Reaction or serology testing for SARS-CoV-2. We excluded patients without a laboratory-confirmed diagnosis of COVID-19 or those without a hospital stay of at least 24 h. The study was approved by HCFMUSP Institutional Review Board, registered under CAEE 39744120.3.0000.0068, and informed consent was obtained from all participants.

### 2.2. Study design

This cross-sectional evaluation of a cohort of COVID-19 survivors is based on a follow-up battery of tests conducted any time between 3 and 11 months after hospital discharge with long COVID. Participants were recruited and assessed between October 2020 and April 2021 in a single cross-sectional time point, and all data was registered within the Research Electronic Data Capture (REDCap) platform. This publication is an ancillary report of a significant multidisciplinary prospective observational project, and details are available at Busatto et al. (22).

### 2.3. Assessments

All data was collected at HCFMUSP premises, a tertiary university hospital, and some questionnaires were administered by teleconsultation before in-person assessments. Sociodemographic and clinical information on age, sex, comorbidities (systemic hypertension, diabetes, obesity, chronic obstructive pulmonary disease, and asthma), symptoms upon hospital admission, and length of hospital stay (LOS) were collected during hospitalization. Inflammatory data was assessed by the C-reactive protein and D-dimer serum concentrations. Time from hospital discharge until the face-to-face assessments were measured in months.

According to the study protocol, functioning and quality of life assessments used a large set of tools and scales (22). Participants were assessed with 1 min Sit to Stand Test; EuroQoL-5 Dimensions-5 Levels (EQ-5D-5L), World Health Organization Disability Assessment Schedule (WHODAS 2.0); Post-COVID-19 Functional Status (PCFS); Functional Assessment of Chronic Illness Therapy—Fatigue (FACIT); Medical Research Council sum score (MRC); Borg Dyspnea Scale, MRC Dyspnea score, Visual Analogue Scale for pain (VAS); Epworth Sleepiness Scale; Insomnia Severity Index; Functional Independence Measurement (FIM); and Functional Oral Intake Scale.

Handgrip strength measurement–used a Jamar^®^ hydraulic hand dynamometer (Sammons Preston, Bolingbrook, IL, USA). Participants were seated with their elbows by their sides, bent at the right angle, and in a neutral wrist position. Each hand was tested three times, and mean scores were recorded. The mean score from the side with the highest results was included for data analysis, as suggested by Budziareck et al. (23), who reported that the strongest is usually the dominant member (23). A G-Walk^®^ inertial sensor (BTS Bioengineering and LetSense Group, Padua, Italy) measured and informed the Timed Up and Go (TUG) results.

**FIGURE 1:**
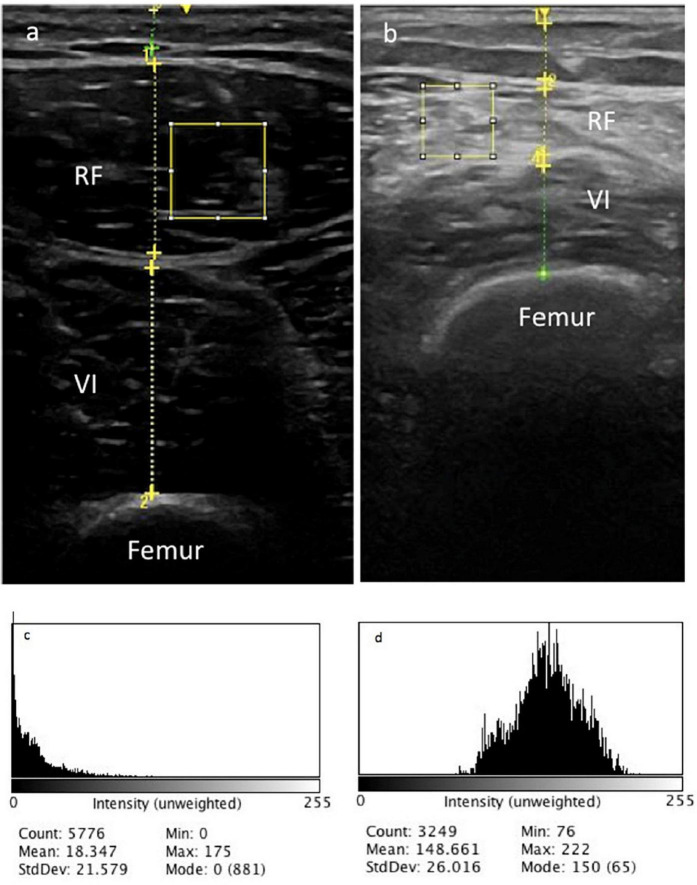
Muscle thickness of right *rectus femoris* (RF) and *vastus intermedius* (VI) muscles ultrasonography of two different long COVID patients **(a,b)**. The selected area represents the region of interest (ROI). Histogram of RF echo intensity of patient a **(c)** and patient b **(d)**.

A portable US system evaluated the MT of the right *rectus femoris* and *vastus intermedius* muscles, the EI of the right *rectus femoris* muscle, and the subcutaneous fat thickness (Figure 1). FujiFilm SonoSite M-Turbo (Bothell, WA, USA) is a robust device, and the parameters were system B-mode broadband linear transducer 6–13 MHz frequency, 6 cm depth, and automatic gain without depth adjustments.

After a 10 min resting period (21), patients were lying in a supine position, with the knee extended and relaxed in a neutral position. For the anatomical landmark, we used the distal third of the distance between the anterior-superior iliac spine and the proximal board of the patella measured by a measuring tape (9, 12), registering a single transversal image with minimal pressure to avoid muscle compression (12, 18, 24). The US tilt was controlled through previous training sessions of all assessors.

We measured MT in centimeters, by the perpendicular distance between the superior and inferior muscle fascia, at the largest diameter without the surrounding fascia, subcutaneous tissue, and skin (9).

Right *rectus femoris* EI was analyzed with the ImageJ^®^ open-access software 1.53a (NIH, Bethesda, MD, USA). The *rectus femoris* region of interest (ROI) included the 2 cm^2^ area (Figure 1a) or as much as possible without any surrounding bone or fascia, calculated by the histogram function of the software. If the limit area was smaller than 2 cm^2^ (Figure 1b), the largest square area was examined without any surrounding bone or fascia.

The ImageJ^®^ software works with an 8-bit resolution and expresses values between “0” (black) and 255 (white) in arbitrary units (AU) (17, 25). MicroDicom viewer software (Version 3.7.7, 64 bit) converted the raw image extracted from the US device. The thickness of the subcutaneous fat tissue was also measured in centimeters and used as a controlling artifact for EI. We used correcting equations to control the artifact effect of the subcutaneous fat tissue on the EI (26).

### 2.4. Data analysis

Muscle morphology of MT and EI were described as means, standard deviations (SD) and 95% confidence intervals (95% CI) for the muscle characterization of patients of long COVID. Then, the morphology variables were tested in association with the demographical, clinical, functional, and quality of life assessments. MT and corrected EI were considered dependent variables, and the demographical, clinical, functional, and quality of life were independent variables.

The statistical analysis was also comprised of stepwise multiple regression in which the demographic, clinical, and comorbidities variables (systemic hypertension, diabetes, obesity, Chronic obstructive pulmonary disease, and asthma) were firstly tested one by one with simple linear regression. The dependent variables, *rectus femoris* and *vastus intermedius* MT, and right *rectus femoris* EI were tested separately for each simple linear regression, and the independent variables that generated a *p*-value below 0.10 were retained for a stepwise multiple linear regression.

The stepwise multiple regression generated a regression model that best explained the dependent variables by adding and subtracting explanatory variables to the model. Each variable added or removed may change the adjusted r^2^ of the model itself and the partial β-coefficient of the remaining independent variables. Therefore, in the regression model in which the explanatory variables generate the largest Adjusted-r^2^, statistical significance (α ≤ 0.05) was retained. Finally, the authors judged the partial β-coefficients 95% confidence interval according to their influence over the dependent variables and excluded the confounding variables, i.e., those who modified the adjusted-r^2^ but had no significance within the regression model. The regression models with the largest Adjusted-r^2^, whose independent variables presented meaningful β-coefficients, statistical significance, and non-confounding characteristics, were presented as our results (27).

A final analysis compared the mean MT of *rectus femoris* and *vastus intermedius* with Student’s *T*-test.

All statistical analysis was conducted with Stata14^®^, and the statistical significance was considered if *p* < 0.05. Given the number of patients included in this study, we applied the central limit theorem and considered that the data could be analyzed with parametrical statistics. We did not input values for the missing data, and all the analysis was conducted per protocol. The researcher who conducted the statistical analysis did not participate in data collection.

### 2.5. Publication guidelines

This study was published according to the STROBE guidelines (28).

## 3. Results

We included 312 patients in this study from a list of 3,751 patients. The participants were mostly males (53.53%), aged 54.59 years (SD 13.50), with mean BMI of 31.77 (SD 13.77). The ultrasound assessments demonstrated the mean muscle thickness values for the rectus *femoris* and *vastus intermedius* of 1.18 cm (SD 0.40) and 1.31 cm (SD 0.49), respectively. These data and other demographic, clinical, and functional characteristics are shown in Table 1. Table 2 presents the results of the quality-of-life evaluations using the EQ-5D-5L.

**TABLE 1:** Demographic, clinical, inflammatory, and functional characteristics.

Variable	Distribution^‡^
Age (years)	54.59 (13.50)/53.09–56.09
Male, *n*; %	167 (53.53%)
	
Body mass index (kg/m^2^)	31.77 (13.77)/30.16–33.37
	
Length of hospital stay (days)	26.80 (21.17)/24.44–29.16
Length of hospital stay at ICU (days)	14.66 (14.07)/13.08–16.24
Intubation, *n*; %	224 (71.79%)
Intubation time (days)	11.69 (9.19)/10.35–13.02
D-dimer concentration (ng/ml)	550.27 (507.22)/514.48–586.06
C-reactive protein (mg/L)	7.72 (18.02)/6.46–8.98
Time since hospital discharge (months)	6.72 (1.38)/6.56–6.88
**Comorbidities**	
Systemic hypertension, *n*; %	180 (57.69%)
Diabetes, *n*; %	114 (36.54%)
Obesity, *n*; %	74 (23.79%)
COPD, *n*; %	15 (4.81%)
Asthma, *n*; %	7 (2.24%)
WHODAS-2	20.45 (8.33)/19.51–21.38
PCFS	1.26 (1.07)/1.15–1.39
FACIT-Fatigue	12.64 (9.22)/12.00–13.67
MRC Dyspnea score	1.07 (1.13)/0.94–1.20
Visual Analog Scale (cm)	4.18 (3.09)/3.83–4.53
Medical Research Council total score	53.00 (7.22)/52.18–53.81
Right knee extensor strength (MRC)	4.54 (0.78)/4.45–4.62
1 min Sit to Stand test (reps)	17.97 (6.90)/17.13–18.80
Borg Dyspnea scale	3.43 (0.14)/3.15–3.70
Timed Up and Go (seconds)	12.77 (5.95)/12.08–13.46
TUG female subgroup	13.77 (6.30)/12.69–14.86
TUG male subgroup	11.92 (5.50)/11.05–12.79
Handgrip strength (kgf/cm^2^)	27.38 (11.77)/26.04–28.71
Females below 40 y.o.	22.37 (8.20)/19.19–25.55
Females from 40 to 59 y.o.	19.19 (7.12)/27.42–20.97
Females above 60 y.o	17.25 (6.03)/15.52–18.98
Males below 40 y.o.	48.59 (8.62)/44.166–53.02
Males from 40 to 59 y.o.	35.34 (9.26)/33.21–37.47
Males above 60 y.o.	30.35 (7.97)/28.42–32.28
Epworth Sleepiness scale	8.31 (5.45)/7.69–8.62
Insomnia Severity Index	6.85 (5.4)/6.21–7.78
Functional Independence Measure	113.32 (13.29)/111.83–114.81
Functional Oral Intake Scale	6.92 (0.44)/6.87–6.97
*Rectus femoris* thickness (cm)	1.18 (0.40)/1.14–1.23
*Vastus intermedius* thickness (cm)	1.31 (0.49)/1.25–1.36
Subcutaneous fat thickness (cm)	1.15 (0.64)/1.08–1.23
Echo-intensity (au)	53.71 (17.44)/51.64–55.78

^‡^Mean, standard deviation (SD), and 95% Confidence interval for continuous variables; *n*, number; %, percentage for categorial variables; ICU, intensive care unit; COPD, chronic obstructive pulmonary disease; WHODAS, World Health Organization Disability Assessment Schedule; PCFS, Post-COVID-19 Functional Status Scale; MRC, Medical Research Council; reps, repetitions; FACIT, Functional Assessment of Chronic Illness Therapy—Fatigue; y.o, years old; au, arbitrary units, ranging from 0 to 255.

**TABLE 2:** Results of the EQ-5D-5L assessment.

Domain	No problems	Slight problems	Moderate problems	Severe problems	Extreme problems
Mobility	150; 48.86%	70; 22.80%	54; 17.59%	29; 9.45%	4; 1.30%
Self-care	216; 70.36%	49; 15.96%	30; 9.77	8; 2.91	4; 1.30
Usual activities	155; 50.49%	74; 24.10%	47; 15.31%	25; 8.14%	6; 1.95%
Pain/discomfort	94; 30.62%	66; 21.50%	85; 27.69%	57; 18.57%	5; 1.63%
Anxiety/depression	121; 39.54%	79; 25.82%	46; 15.03%	56; 18.30%	4; 1.31%

Data are presented as *n*; %.

### 3.1. Muscle thickness

During the simple linear regression analysis, no correlation was found between *rectus femoris* thickness and the total length of hospital stay, the event of intubation, asthma, diabetes, pain intensity, time since discharge, and C-reactive protein levels. Hence, these variables were not included in the multiple regression modeling analysis.

The stepwise multiple regression for the predictors of the *rectus femoris* MT started adding demographical and clinical variables. In this first step, age, sex, the presence of COPD, and systemic hypertension were tested in combination. COPD and systemic hypertension did not contribute to the model (*p* = 0.282 and *p* = 0.920, respectively), being removed from the model. The second step added the quality-of-life variables (EQ-5D-5L and WHODAS 2.0) and the functional scales (PCFS and FACIT), however, there was no contribution to the regression model (*p* = 0.399; *p* = 0.264; *p* = 0.633; *p* = 0.467, respectively).

The fourth step added and tested the functional assessments (MRC total score, MRC for the right knee extension, 1 min Sit to Stand Test, TUG, handgrip strength, pain intensity, Epworth sleepiness scale, Insomnia Severity Index, Functional Oral Intake Scale, and FIM). The 1 min Sit to Stand Test, TUG, Functional Oral Intake Scale, FIM, Insomnia Severity Index, and the MRC for the right knee extension, in combination with the other variables, especially the MRC total score, did not contribute to the model (*p* = 0.917; *p* = 0.969; *p* = 0.180; *p* = 0.465, *p* = 0.697, and *p* = 0.516, respectively). The variables that generated the best model during the fourth step caused the sex variable to be removed from the model, as it became non-significant (*p* = 0.912). After this step, the remaining variables were age, pain, MRC total score, handgrip score, and Epworth sleepiness scale score.

The last step included the subcutaneous fat thickness, D-Dimer levels, C-Reactive Protein levels, and the time gap since hospital discharge. The time since hospital discharge and C-Reactive Protein levels did not contribute to the model and were removed (*p* = 0.229 and *p* = 0.210, respectively). The remaining variables of the model with the best adj-r^2^ after the final step were age, pain intensity, MRC total score, handgrip strength, Epworth sleepiness scale, subcutaneous fat thickness, and D-Dimer levels.

Pain intensity, MRC total score, and Epworth sleepiness scale had partial *p*-values above 0.05. Therefore, we ran a likelihood ratio test comparing both models, with and without these three variables. This test showed that the largest model was the best (Likelihood-ratio test, *p* = 0.004), therefore the three variables were not removed. Finally, the model was analyzed for homoscedasticity with the Breusch-Pagan/Cook-Weisberg test for heteroscedasticity, being considered homoscedastic (*p* = 0.182).

By analyzing the β-coefficients, pain intensity, MRC total score, and D-Dimer levels were considered confounders and removed from the final model. Table 3 presents the best regression model for *rectus femoris* MT.

**TABLE 3:** Best regression model for muscle thickness prediction of *rectus femoris* and *vastus intermedius*.

	β	95% CI		*P*-value	r^2^; Model *P*-value
***Rectus femoris***					
Age	-0.006	−0.009	−0.003	<0.001	27.51%; *p* < 0.0001 adj-r^2^ = 0.264
Handgrip strength	0.014	0.010	0.018	<0.001	
Epworth Sleepiness scale	0.008	0.001	0.016	0.032	
Subcutaneous fat thickness	0.112	0.040	0.184	0.003	
***Vastus intermedius***					
Age	-0.007	−0.012	−0.004	<0.001	21.12%; *p* < 0.0001 adj-r^2^ = 0.194
Pain intensity	0.199	0.117	0.281	0.035	
Handgrip strength	0.005	0.045	0.000	0.045	
Epworth Sleepiness scale	0.011	0.002	0.02	0.020	
FIM	0.007	0.002	0.013	0.012	
Time since hospital discharge	0.046	0.008	0.084	0.018	

β, linear regression beta coefficient; CI, confidence interval; FIM, Functional Independence Measure; adj-r^2^, adjusted-r^2^.

Regarding the *vastus intermedius* MT, the same steps were followed. The single regression analysis showed that total length of hospital stay, intubation, systemic hypertension, asthma, and subcutaneous fat thickness were not correlated with *vastus intermedius* MT (*p* = 0.291; *p* = 0.354; *p* = 0.203; *p* = 0.480; and *p* = 0.492, respectively), and were not considered in the stepwise regression analysis.

In the first step, age, sex, presence of COPD, and diabetes were tested. The presence of diabetes did not contribute to the model (*p* = 0.293), being removed from the model. The second step added WHODAS 2.0, EQ-5D-5L, PCFS, and FACIT scores. The EQ-5D-5L, PCFS, and FACIT scores did not contribute to the model and were removed (*p* = 0.722, *p* = 0.484, and *p* = 0.687, respectively). The third step added MRC total score, MRC for the right knee extension, 1 min Sit to Stand Test, TUG, handgrip strength, pain intensity, Epworth sleepiness scale, Insomnia Severity Index, Functional Oral Intake Scale, and FIM. This analysis demonstrated that MRC for the right knee extension, MRC total score, 1 min Sit to Stand Test, TUG, WHODAS 2.0, presence of COPD, Insomnia Severity Index, and Functional Oral Intake Scale did not contribute to the model and were removed (*p* = 0.378; *p* = 0.355; *p* = 0.791; *p* = 0.224; *p* = 0.260; *p* = 0.300; *p* = 0.473; and *p* = 0.124, respectively). During this step, the presence of sex was removed, as it became not-statistically significant (*p* = 0.681).

The last step included the time gap since hospital discharge, subcutaneous fat thickness, D-Dimer levels, and C-Reactive Protein levels. D-Dimer, C-Reactive Protein levels, and subcutaneous fat thickness did not contribute to the model and were removed (*p* = 0.417, *p* = 0.875, and *p* = 0.228, respectively). The remaining model consisted of age, presence of COPD, pain, handgrip strength, Epworth sleepiness scale score, FIM, and time gap since hospital discharge as the best predictors of *vastus intermedius* MT. Even though the presence of COPD contributed to the adj-r^2^, it had questionable significance (*p* = 0.275). Therefore, a Likelihood-ratio test was conducted comparing the regression models with and without the COPD variable, showing that the presence of COPD may be removed from the model without jeopardizing its predictive capacity (*p* = 0.268. Oppositely from the *rectus femoris* analysis, the final model is not homoscedastic (*p* = 0.002, Breusch-Pagan/Cook-Weisberg test for heteroscedasticity) and should be considered with caution.

The β-coefficient analysis shows that age, handgrip strength, and FIM are the least clinically significant. The final predictive model of the *vastus intermedius* thickness is also presented in Table 3.

Finally, the mean MT of the *rectus femoris* and *vastus intermedius* were significantly different (1.17 cm, SD 0.40 and 1.29 cm SD 0.47 cm, respectively; *p* < 0.0001).

### 3.2. Echo-intensity

Initially, raw values of EI were corrected to subcutaneous fat thickness, according to Young et al. (26). The steps for analyzing the EI were followed similarly to those for the MT analysis, and the results of simple regression tests evidenced that total length of hospital stay, the event of intubation, presence of COPD, asthma, diabetes, and Epworth Sleepiness Scale score were not associated with right *rectus femoris* EI (*p* = 0.518, *p* = 0.989, *p* = 0.265, *p* = 0.626, *p* = 0.634, and *p* = 0.699, respectively), and were not considered in the stepwise regression analysis.

The first step included sex, age, quality of life and level of disability scales (WHODAS 2.0 and EQ-5D-5L), COVID-19 functional scale, and fatigue scale. The quality of life, PCFS, and FACIT scales did not contribute to the model and were removed (*p* = 0.185, *p* = 0.212, and *p* = 0.823, respectively), whereas sex, age, and level of disability remained. The second step added pain, TUG, MRC total score, MRC for the right knee extension, handgrip strength, Insomnia score, FIM, and Functional Oral Intake Scale. During the second step, pain, Insomnia score, FIM, Functional Oral Intake Scale, MRC for the right knee extension, and MRC total score did not contribute to the model and were removed (*p* = 0.740; *p* = 0.969; *p* = 0.674; *p* = 0.535; *p* = 0.546; and *p* = 0.986, respectively). The third and last step added the subcutaneous time gap since hospital discharge, fat thickness, D-Dimer levels, and C-Reactive Protein levels, and the analysis evidenced that the subcutaneous fat thickness and D-Dimer levels did not contribute to the model and were removed (*p* = 0.500 and *p* = 0.422, respectively).

The remaining variables with the best adj-r^2^ were age, sex, level of disability, TUG, handgrip strength, time gap since hospital discharge, and C-Reactive Protein levels. However, the variables level of disability and the time gap since hospital discharge had partial *p*-values above 0.05. A Likelihood-ratio test was conducted comparing the regression models with and without these variables, showing that the simplest model is more accurate than the complex one (*p* = 0.078).

The final model is homoscedastic (*p* = 0.758, Breusch-Pagan/Cook-Weisberg test for heteroscedasticity) (29, 30), as demonstrated in Table 4.

**TABLE 4:** Best regression model for *rectus femoris* (RF) corrected (24) echo-intensity prediction.

	β	95% CI		*P*-value	r^2^; Model *P*-value
Sex (male)^‡^	-37.04	−43.40	−30.67	<0.001	44.39%; p < 0.0001 Adj-r^2^ = 0.43
TUG	1.175	0.638	1.712	<0.001	
Epworth Sleepiness scale	0.640	0.064	1.216	0.030	
C-Reactive protein levels	0.465	0.176	0.754	0.002	

RF, *rectus femoris*; β, linear regression beta coefficients; CI, confidence interval; TUG, Timed Up and Go; adj-r^2^, adjusted-r^2^. ^‡^Male patients generate a β coefficient of -37.07 in the regression model.

## 4. Discussion

We assessed right *rectus femoris* and *vastus intermedius* MT and *rectus femoris* EI in long COVID patients 3–11 months after hospital discharge. A combination of demographic, clinical, functional, and inflammatory parameters were associated with the quantitative (MT) and qualitative (EI) characteristics of the *rectus femoris* and *vastus intermedius* muscles.

We demonstrated similar demographic correlations for both *rectus femoris* and *vastus intermedius* MT, including significant negative correlations of age in both *rectus femoris* and *vastus intermedius* MT. Interestingly, however, was the influence of different clinical and functional independent variables in the *rectus femoris* and the *vastus intermedius* MT. Thicker *rectus femoris* was associated with younger ages, greater muscle strength, less daytime sleepiness, and thicker subcutaneous fat thickness (r^2^ = 27.51; *p* < 0.0001). On the other hand, thicker *vastus intermedius* was associated with younger age, greater functional independence and muscle strength, higher pain intensity, as well as lower excessive daytime sleepiness, and longer time since hospital discharge (r^2^ = 21.12%; *p* < 0.0001). Lower *rectus femoris* EI was associated with male sex, increased functional mobility, lower excessive daytime sleepiness and inflammation levels (r^2^ = 44.39%; *p* < 0.0001).

### 4.1. Clinical applications

Andrade-Junior et al. (9) also demonstrated decreasing MT and loss of quadriceps quality in 32 critically ill COVID-19 patients during hospitalization in intensive care units [de Andrade-Junior et al. (9)]. Using the same US methodology, we confirmed their results in long COVID patients regarding reduced MT in the *rectus femoris* and *vastus intermedius* muscles and altered EI in the *rectus femoris* muscle. We agree that prolonged immobilization, use of steroids, neuromuscular blocking agents, electrolytic imbalances, systemic inflammatory metabolic status, and COVID-19 severity may have influenced their results (9). Surprisingly, qualitative and quantitative muscle changes may persist 3–11 months after hospital discharge and not recover spontaneously, despite the absence of the influencing factors of acute hospitalization. On the other hand, other factors, including physical inactivity exacerbated by quarantine, social distancing measures, poor nutritional status, environmental exposure to air pollution, and poor socioeconomic status may play a role in maintaining the qualitative characteristics in the skeletal muscles of long COVID patients (31). Also, it is meaningful to consider that the progressive skeletal muscle wasting observed in the first days of ICU stay (10) has not been managed with specific interventions in our patient population.

#### 4.1.1. Muscle thickness

In a previous study, when we evaluated post-COVID-19 patients, in the post-acute phase, in transition from hospitalization in the ICU to the hospitalization unit for intensive rehabilitation, the patients had severe and generalized muscle weakness (MRC: 43.81 ± 7.76) (32). The recovery of muscle strength and other clinical parameters, on average, after 22 days of hospitalization, allowed improvement of functioning and rehabilitation hospital discharge with goals achieved (32). In that study, FIM scores played a predictive role in the duration of an intensive inpatient rehabilitation protocol immediately after hospital discharge (32). However, even with normal MRC sum scores (MRC: 52.92 ± 7.18), our long COVID patients still showed decreased MT and function, measured by the 1 min Sit to Stand Test and TUG, and poor handgrip strength. Different from Andrade Junior et al. (9), we found a significant association between handgrip strength and *rectus femoris* and *vastus intermedius* MT in long COVID patients. Our findings agree with the recent data presented by Damanti et al. (33), who also identified reduced handgrip strength as significantly associated with lower medial gastrocnemius MT among COVID-19 survivors one month after hospital discharge. This is a relevant finding, as we demonstrate that possible muscle changes are still present in long COVID patients, a finding not previously described in the literature.

On the other hand, greater functional independence, was associated with reduced *vastus intermedius* MT, but not with the *rectus femoris* MT. We hypothesize that different components of the quadriceps muscle may receive influence from different functional demands. As we have evaluated long COVID patients after hospital discharge, additional muscle mass deterioration could not be excluded due to sedentary behavior imposed by containment measures (8). Our findings suggest that specific assessments, including the early US, to confirm the presence of existing skeletal muscle wasting should be considered in long COVID patients. Daily sessions of neuromuscular electrical stimulation over the *vastus medialis* and *vastus lateralis* muscles for seven consecutive days in the ICU setting improved muscle strength and functionality with a potential protective effect on muscle mass loss of critical COVID-19 associated with sepsis and septic shock (34, 35). A 6-week course of respiratory training, upper limb straightening, and aerobic exercises also increased *rectus femoris* and *vastus intermedius* MT and reduced *rectus femoris* and *vastus intermedius* EI in a series of post-COVID-19 (36). Our findings suggest that rehabilitation interventions may also be considered in long COVID patients after hospital discharge, targeting improving skeletal muscle quantity and quality.

Other specific interventions, such as strengthening training, demonstrate positive changes in MT of the quadriceps muscle of non-COVID-19 people (37). Therefore, early detection of muscle quality and thickness changes may guide specific rehabilitation interventions in long COVID-19.

Sabatino et al. (13) reported low MT of the *rectus femoris* and *vastus intermedius* in a critically ill non-COVID-19 hospitalized population using a similar US technique, showing *rectus femoris* measures similar to our findings but thinner *vastus intermedius* (13). Similar to our findings, Paris et al. (21) also identified thinner MT in older people (21). Therefore, MT could be explained by aging (38). Only a few studies were identified in a literature search on normative MT data collected with the same technique we used in our study (39, 40).

We identified one study that reported MT of the *rectus femoris* and *vastus intermedius* using this technique in a non-COVID-19 hospitalized population (13). The measurement of the *rectus femoris* was similar to our population; the *vastus intermedius* in our population was slightly thicker (13). Interestingly, we observed statistically significant greater *vastus intermedius* MT values than *rectus femoris* (*p* < 0.001). A possible explanation for our observation of greater *vastus intermedius* MT may be the heterogeneous aging-related muscle atrophy in the different portions of the quadriceps femoris muscle (41, 42). Significantly higher reductions in *rectus femoris* contractile and non-contractile muscle elements compared to the *vastus intermedius* have already been described in males and females (41, 42).

Interestingly, higher values of *vastus intermedius* MT and not *rectus femoris* MT were significantly associated with a more extensive period since hospital discharge. This finding may also suggest that *vastus intermedius* MT may recover over time. Future cohort studies should confirm this finding. Unfortunately, previous US studies captured combined measurements of *rectus femoris* and *vastus intermedius* muscles (9, 10), which were unable to identify this feature in acute COVID-19 patients. In any case, this interesting finding may suggest that future studies should assess *rectus femoris* and *vastus intermedius* separately, as we have performed in our study. This report has relevant clinical implications, as different therapeutic interventions or dosing of the same interventions may be indicated to manage reduced *rectus femoris* and *vastus intermedius* MT.

Muscle weakness and fatigue are among the main complaints in long COVID patients (2–5). Our data demonstrated that muscle strength, traditionally used in clinical settings, lies within the normal range in our hospitalized long COVID patients, with a narrow 95% confidence interval.

Handgrip dynamometry is a sensitive tool to measure muscle strength during rehabilitation (43), an overall assessment physical of muscle strength and capacity (44, 45). Our results show that those with weaker handgrip strength had lower *rectus femoris* and *vastus intermedius* MT. This important finding suggests that handgrip strength may inform specific rehabilitation interventions for restoring muscle quantity in long COVID patients, a subject not yet well-explored in the literature.

The prevalence of sarcopenia is estimated to be about 48.0% (21 studies, including 5,407 patients) and is more likely to affect ICU patients (46). Despite the high heterogeneity, this recent meta-analysis of pooled data may explain the findings identified in our study, composed mainly of patients who were treated in the ICU. US assessments can detect progressive skeletal muscle wasting during the ICU stay of severely affected COVID-19 patients (9, 10). As a non-invasive, low-cost, portable, non-ionizing, and safe technique, US devices will allow clinicians to detect possible muscle changes that can benefit further assessments and interventions in long COVID patients. Besides already well-established factors that influence MT, such as age, our results demonstrated that the functional status assessed by the Handgrip strength influenced both *rectus femoris* and *vastus intermedius* MT.

We were also unable to capture associations between MT and comorbidities and duration of hospital stay, significant findings different from those identified in COVID-19 survivors one month after hospital discharge (33). One possible explanation is that different skeletal muscles with different functional and biomechanical demands may present different biological responses. Interestingly, our multivariate model significantly associated higher daytime sleepiness assessed by the Epworth Sleepiness scale with larger MT at the *rectus femoris* and *vastus intermedius* muscles. We were unable to identify similar reports in the literature. More studies should be performed to confirm this striking finding. Likewise, higher pain scores were significantly associated with thicker *vastus intermedius*, which requires further investigation.

#### 4.1.2. Echo-intensity

*Rectus femoris* muscle quality should be assessed using US EI in long COVID patients after hospital discharge. We have demonstrated that qualitative skeletal muscle characteristics captured by *rectus femoris* corrected EI may be different in two long COVID patients of the same sex and age, presenting with fatigue and weakness yet, with grade 5 knee extensor strength during physical examination, as demonstrated in Figures 1a, b. Similar to previous reports, we also found a significant positive correlation between the male sex and lower *rectus femoris* EI (39, 47). Our findings agree with reports from several authors of EI as a surrogate measure of muscle quality (15, 26, 48). The enhanced EI represents changes in muscle quality; higher EI is associated with increased intramuscular adipose and fibrous tissue (49–51).

Our protocol for ROI selection was based on previous studies conducted by Vieira et al. (17), and Cartwright et al. (17, 25). EI measurements obtained by whole muscle image have higher reliability than a smaller, constant-size rectangular ROI (52). However, due to the smaller areas identified in several of our patients, we decided to use a constant-size ROI.

Our results identified similar well-established EI influencing factors, such as sex (39, 53). Different from findings from other authors (39, 54), age has not influenced our multivariate model. Even though the 1 min stand-up test has also not played an influence, like in previous studies (55–57), we have identified the influence of the TUG on the EI values. Our results showed that higher *rectus femoris* EI is associated with longer TUG values (β = 1.175, *p* < 0.0001).

Few studies have examined the relationship between EI and TUG. This exact correlation has also been described in non-COVID patients (58). Higher EI of the *vastus lateralis* and gastrocnemius muscles were associated with longer TUG values (58). However, we could not identify any other studies associating EI and TUG in COVID-19 patients.

EI predicts functional outcomes (59, 60), including mobility and gait-related activities. For instance, an EI analysis of the quadriceps muscle, assessed by a 30 s sit-up test with the non-active elderly, was considered the best predictor of functional performance (55, 61). This evidence was also found among older adults in several publications (r = –0.49 to –0.56) (55–57). *Rectus femoris* EI significantly correlates with the 30 s sit-up test and walking speed. Even though we did not identify an association between *rectus femoris* EI with age, similar findings were also present in our study, as we demonstrated a positive correlation between *rectus femoris* EI and TUG in long COVID patients. As these associations may arise from aging (56, 57), future studies should clarify whether COVID-19 alone interferes with muscle EI and whether specific interventions may revert this finding. Proper functional mobility is a key element for functional autonomy and a healthy lifestyle, especially in the elderly population. We highlight the relevance of the metabolic cost, as well as the mechanical work and efficiency during walking (62, 63). The influence of such parameters should also be assessed in future long COVID studies.

Little is known about the effects of long COVID on the skeletal muscles and their functional status. Our findings suggest the influence of the male sex, inflammatory status, and functional scales with the altered EI seen on the *rectus femoris* US imaging. Persistently elevated blood levels of C-reactive protein seem to be associated with long COVID symptoms (22). Augmented levels of serum C-reactive protein may also be related to the severity of persistent long COVID symptoms, including fatigue, poor physical performance, dyspnea, and psychiatric changes (64). The influence of C-reactive protein and other inflammatory biomarkers has already been described in COVID-19 survivors (65–68).

Interestingly, our results also evidenced the significant influence of C-reactive protein on the multivariate model for the prediction of corrected EI of the right *rectus femoris* muscle. This innovative finding may offer the rationale for a persistent inflammatory mechanism also involved in skeletal muscle composition, fatigue, and muscle weakness (67). Indeed, skeletal muscle function and quality assessment may also represent a valuable prognostic tool in long COVID patients (69).

Similar to the findings for MT, we have also identified the influence of higher daytime sleepiness on higher *rectus femoris* EI values in our multivariate model. We were unable to identify similar reports in the literature. More studies should be performed to confirm this finding.

To our knowledge, this is the largest cohort of COVID-19 patients assessed during in-person visits, 3–11 months after hospital discharge, including a higher percentage of patients who required intensive care and mechanical ventilation. Muscle weakness in the long term has been described previously. However, to the best of our knowledge, we are the first to report a quantitative measure of this finding 3–11 months after hospital discharge. We emphasize the relevance of continuous follow-up of these patients (33, 46). This US protocol can be widely used as a screening test for early detection of skeletal muscle changes in long COVID patients.

### 4.2. Limitations

A significant limitation of our study is the lack of a control group to compare our findings. Ethical and sanitary issues faced during the COVID-19 pandemic did not allow healthy individuals in our hospital facility. We were not able to recruit a control group of patients with other health conditions as admissions for such individuals have substantially reduced during the COVID-19 pandemics, and a historical control or a control group from a different time period could also have the influence of a sedentary lifestyle after the end of the same period. Regardless of the described shortcomings, our results have positive clinical implications as US findings help identify long COVID patients with the potential to benefit from interventions to increase MT and reduce EI. This study has exploratory and hypothesis-generating characteristics, and we aimed to provide clinicians with a straightforward methodology to detect potential changes as a screening tool for further and more complex investigations.

We were unable to perform three US images from the same patient, nor calibrate images during the analysis process given time constraint to allow us to examine the large number of patients in a concise time in restricted settings. Nonetheless, we believe our results are not compromised, given the uniform training given by one of the researchers with long-lasting years of experience (C.A.R.P.A.).

We have not assessed MT and EI prior to COVID-19 infection or during hospitalization. Therefore, we cannot identify the direct effects of COVID-19 in the skeletal muscle of long COVID patients.

A final limitation is that we could not differentiate fat from fibrous tissue. The analysis of the histology of the *rectus femoris* with a non-coaxial muscle biopsy, at the same point as the US analysis, would facilitate the understanding of the pathophysiological process of our findings. Biopsy or resonance imaging can be carried out to clarify this issue further.

## 5. Conclusion

Muscle thickness of the *rectus femoris* and *vastus intermedius* and EI of the *rectus femoris* may be abnormal in patients with long COVID 3–11 months after hospital discharge. Also, male sex, TUG, Epworth Sleepiness scale, and the C-Reactive Protein were predictors for EI. *Rectus femoris* MT is associated with age, handgrip strength, Epworth Sleepiness Scale, and subcutaneous fat thickness. *Vastus intermedius* MT is associated with age, FIM, pain intensity, handgrip strength, Epworth Sleepiness scale, and time since hospital discharge in long COVID patients.

These findings highlight the importance of suitable tools to capture individual rehabilitation needs. In the same way, the follow-up of this population must identify, monitor, and manage future outcomes, such as the possibility of complications with falls and subsequent fractures, to proper rehabilitation.

## Data availability statement

Requests for materials and datasets should be addressed to the corresponding author.

## Ethics statement

This study involves human participants and was reviewed and approved by HCFMUSP Institutional Review Board Hospital das Clinicas da Faculdade de Medicina da Universidade de São Paulo, registered under CAEE 39744120.3.0000.0068. The patients/participants provided their written informed consent to participate in this study.

## Author contributions

MI and LRB contributed to conceptualization, methodology, and visualization. MI, SSTU, GSN, CARPA, and LRB contributed to data curation. ACAS contributed to the formal analysis. MI, SSTU, GSN, ARM, and CARPA contributed to the investigation. MI contributed to project administration. LRB contributed to supervision and validation. MI, SSTU, GSN, ARM, and ACAS wrote the original draft. MI, SSTU, GSN, ACAS, and LRB reviewed and edited the manuscript. HCFMUSP COVID-19 Study Group contributed to conceptualization and data curation. All authors contributed to the article and approved the submitted version.
